# Introducing the UK Antimicrobial Registry (UKAR) study: providing real world data on new antimicrobials to support antimicrobial stewardship and tackle antimicrobial resistance

**DOI:** 10.1093/jacamr/dlae107

**Published:** 2024-07-19

**Authors:** Jacqueline Sneddon, Gary J Macfarlane, Gareth T Jones, Laura Moir, Rebecca Parr, David Jenkins, Tracey Guise, Jonathan A T Sandoe, R Andrew Seaton, UKAR Steering Committee, Nicholas Brown, Christopher Longshaw, Fran Garraghan

**Affiliations:** British Society for Antimicrobial Chemotherapy, Birmingham, UK; Epidemiology Group, University of Aberdeen, Aberdeen, UK; Epidemiology Group, University of Aberdeen, Aberdeen, UK; Epidemiology Group, University of Aberdeen, Aberdeen, UK; Epidemiology Group, University of Aberdeen, Aberdeen, UK; British Society for Antimicrobial Chemotherapy, Birmingham, UK; Department of Clinical Microbiology, University Hospitals of Leicester NHS Trust, Leicester, UK; British Society for Antimicrobial Chemotherapy, Birmingham, UK; British Society for Antimicrobial Chemotherapy, Birmingham, UK; School of Medicine, University of Leeds, and Leeds Teaching Hospitals NHS Trust, Leeds, UK; British Society for Antimicrobial Chemotherapy, Birmingham, UK; Queen Elizabeth University Hospital, Glasgow, UK

## Abstract

The UK Antimicrobial Registry (UKAR) has been developed to capture data on real world usage of antimicrobial agents with an initial focus on those used to treat drug-resistant infections. Several industry partners have committed support for the study, which is included in the National Institute for Health and Care Research (NIHR) portfolio in England with similar arrangements in the three devolved UK nations. The two antimicrobials in the National Institute for Health and Care Excellence (NICE) subscription model pilot (cefiderocol and ceftazidime/avibactam) are included in the UKAR and future expansion of work in this area is planned. This model decouples payment from usage by using a fixed annual fee. The study will provide information on the characteristics of patients receiving study drugs, the infections being treated, treatment effectiveness and adverse events. UKAR potentially provides a novel resource of enduring value to support healthcare in the UK and more widely and contribute to AMR National Action Plan goals for optimal use of antimicrobials.

## The need for real world data on new antimicrobials

The relative dearth of new antimicrobials over the past few decades means it is critical to fully understand the place of any new treatment options in routine clinical practice. While clinical trials supporting product licences for new antimicrobials provide evidence of safety and effectiveness, tight inclusion and exclusion criteria mean these trials often exclude patients who may benefit from the drugs. Real world data are needed post-licensing to understand the range of clinical use (which may be outside licensed indications) and outcomes of new antimicrobials, particularly in patients with complex, uncommon and drug-resistant infections and in patients with significant comorbidity. A better understanding of how new agents are used will help development and use of innovative approaches to funding such as the NICE value-based subscription model^1^ to support further investment in research and development. This model provides an innovative approach to paying for antimicrobial products by decoupling payments from usage. Instead, manufacturers receive a fixed annual fee based on the health technology assessment of their products’ value to the National Health Service (NHS). The UKAR study^2,3^ has been developed by the British Society for Antimicrobial Chemotherapy (BSAC) in collaboration with investigators from the University of Aberdeen School of Medicine, Medical Sciences and Nutrition to provide prescribers and organizations the opportunity to capture real world data on newly licensed antimicrobial usage and outcomes with the aim of informing their position in clinical practice. While other similar registries have been developed to study the use of specific antimicrobials,^4–8^ to our knowledge this registry study is unique in its aim to prospectively incorporate all newly licensed antimicrobials and to align with the developing novel NICE subscription model.

## Development of the UK Antimicrobial Registry (UKAR) study

UKAR is a collaborative epidemiological study in which the BSAC is working with a team from the Epidemiology Group at the University of Aberdeen, NICE and several industry partners. The study is included in the NIHR portfolio in England with similar arrangements in Scotland, Wales and Northern Ireland. UKAR collects information on the use of recently launched antibiotics, with a particular focus on those used to treat infections due to multi-drug-resistant organisms. The two antimicrobials currently in the NICE subscription model pilot (cefiderocol and ceftazidime/avibactam) are included in UKAR providing valuable additional clinical data to complement supply data collected via NHS England Blueteq forms. The study builds on experience with other antimicrobial registries^4–8^ as well as experience with non-infection related patient registries.^9,10^

## Progress to date

Following development of data requirements, build of the UKAR online database and ethics approval from all UK nations, the study team started working with pilot site hospitals in early 2023. Open recruitment of hospital sites began in summer 2023, providing opportunity for hospitals across the UK to be part of a programme with national and international importance. Each hospital has a principal investigator (PI) with a multi-professional approach taken to encourage clinicians from Infectious Diseases, Microbiology and Pharmacy to take on these roles. The study is also registered with the NIHR Associate PI scheme.^11^

**Figure 1.**
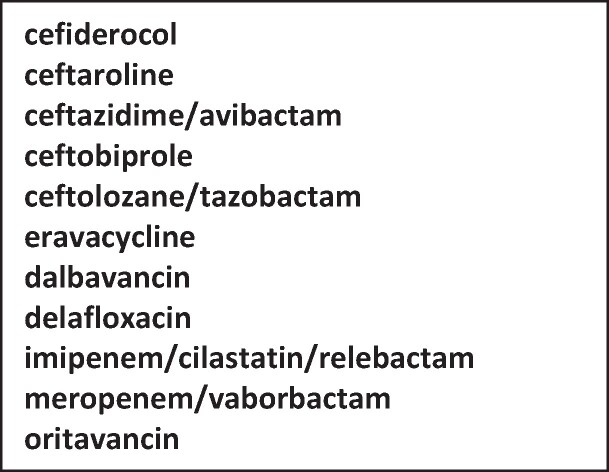
UKAR study drug list 2024.

Adult patients are potentially eligible if they have been prescribed any drug on the eligible drug list within their current stay in hospital unless treatment with the drug is stopped within 24 h. The current study drug list is shown in Figure 1 and inclusion of antimicrobials in the study is independent of any pharmaceutical company involvement in the study. This list is reviewed annually.

Data are collected from the medical records of participants who have given informed consent, with arrangement in place for adults without capacity. Clinical data, including infection site, comorbidity, microbiology isolates and susceptibility, eligible drug and dosing regimens are collected as well as prior and concurrent antibiotic therapy. Data are collected 56 days from start of treatment, 28 days after completion of treatment and follow-up for relapse or recrudescence is conducted at 6 and 12 months. The full UKAR protocol is available online.^12^

## Future ambitions

As of the end of April 2024, the UKAR has 29 participant sites and 240 patients recruited. The goal is to have 1000 participants by May 2026 from a mix of tertiary centres and district general hospitals across the UK.

To date, the UKAR has been supported by three industry partners whose input has been invaluable in shaping the database design and informing reporting. With the future addition of more study drugs including new antifungal agents coming to market, BSAC is actively engaging with other potential industry partners to join the UKAR.

The recently announced expansion of the NHS England NICE de-linkage scheme model^13^ for the evaluation and purchase of all new-to-market antimicrobials will facilitate targeted use in suitable patients at a fixed total cost to healthcare providers. The UKAR is well placed to provide quantitative data on use of future new agents.

An additional aspect of the UKAR study is inclusion of a proof-of-concept virtual registry using data within NHS Scotland’s national Datamart. This parallel project, in collaboration with a team from the University of Strathclyde Institute of Pharmacy and Biomedical Science, will seek to replicate data collected in the hospital database registry and will enable additional questions to be answered by linking clinical data sets held within a secure safe haven. This approach has the advantage of broader inclusion, as de-identified data are extracted and therefore do not require individual patient consent. Data can easily be scaled to investigate use of other antimicrobials rather than just the list of new agents currently within UKAR. As data systems evolve across the other UK nations this approach may be replicated.

Last, the study steering group are actively considering expanding this programme of work to facilitate audit activities and onboarding of existing data sets. Both activities will support an increase in patient recruitment and data collection.

In these times of escalating global antimicrobial resistance^14^ and paucity of data supporting new antimicrobials outside of their licensed indications, the UKAR study will provide essential intelligence relating to patterns of use and associated outcomes. Data outputs will inform the safe and effective use of new antimicrobials in clinical practice, support guideline development and the NICE value-based subscription model for new antimicrobials.

## Transparency declarations

J.S. is an employee of BSAC and Lead for the UKAR Programme. D.J. is President of BSAC. T.G. is Chief Executive Officer of BSAC. J.A.T.S. is a council member of BSAC, a member of the British Society for Allergy and Clinical Immunology Penicillin Allergy working party and a panel member of the European Society for Clinical Microbiology and Infectious Diseases guideline for antibiotic allergy. J.A.T.S. has had research income from NIHR, EPSRC, MRC, Welcome, John Moulton Foundation and Lumix diagnostics. J.A.T.S. has done consultancy work for Tillotts Pharma and Medtronic (2021) to design/deliver educational event and consensus survey, respectively. R.A.S. is Vice President/President elect of BSAC, co-lead of the BSAC OPAT initiative, Chair of the Scottish Antimicrobial Prescribing Group and has advised on the pilot NICE antimicrobial subscription model. R.A.S. has received fees for consultancy work for NAPP/Mundipharm, Pfizer and Advanz and for speaking at educational events for Menarini and Shionogi.
